# Potential Usefulness of Bacteriophages for the Treatment of Multidrug-Resistant *Acinetobacter* Infection

**DOI:** 10.21315/mjms2023.30.5.2

**Published:** 2023-10-30

**Authors:** Fahad Raees, Azian Harun, Abdalla Ahmed, Zakuan Zainy Deris

**Affiliations:** 1 Department of Medical Microbiology and Parasitology, School of Medical Sciences, Universiti Sains Malaysia, Kelantan, Malaysia; 2 Department of Microbiology, College of Medicine, Umm al-Qura University, Makkah, Kingdom of Saudi Arabia; 3 Hospital Universiti Sains Malaysia, Kelantan, Malaysia

**Keywords:** bacteriophage, Acinetobacter baumannii, multidrug-resistance, Gram-negative ‘superbugs’, phage therapy

## Abstract

Bacteriophages were discovered in early 20th century. However, the interest in bacteriophage research was reduced with the discovery of antibiotics. With the increasing number of infections due to multidrug-resistant (MDR) organisms, the potential usefulness of bacteriophages as therapeutic agents has been re-evaluated. In this review, we found that more than 30 lytic bacteriophages that infect *Acinetobacter* species have been characterised. These are mainly members of Caudovirales, with genome sizes ranging from 31 kb to 234 kb and G+C contents ranging from 33.5% to 45.5%. The host range can be as low as < 10% of all tested *Acinetobacter* strains. Fourteen published murine trials indicated positive outcomes in bacteriophage-treated groups. Only two case reports were pertaining to the use of bacteriophages in the treatment of *Acinetobacter* infections in humans; in both cases, the infections were resolved with bacteriophage therapy. The use of bacteriophages has been associated with reduced *Acinetobacter* burden in the environment, as shown in two studies. The major limitation of bacteriophage therapy is its highly selective host strain. In conclusion, the potential usefulness of bacteriophage therapy for the treatment of MDR *Acinetobacter* species has been documented only in limited studies and more research is needed prior to its extensive use in clinical practice.

## Introduction

Bacteriophages (also known as phages) are viruses that infect bacteria. In particular, lytic bacteriophages invade bacterial cells and disrupt bacterial metabolism, which leads to bacterial lysis (1). These viruses are the most abundant organisms on earth, with an estimated 10^31^ phage particles (2). This number is more than the combination of all other organisms on earth. Tailed bacteriophages (order Caudovirales) are among the earliest phages that have been discovered and studied extensively. Although the outcomes of trials during the pre-antibiotic era were inconsistent and non-reproducible, the application of bacteriophages in the treatment of infection indicates their potential usefulness as antibacterial agents (3).

### History of Bacteriophages

As early as 1915, Twort (4) reported white micrococcus colonies that became transparent upon contact with a small portion of one of the other micrococcus glassy colonies. The growth at the contact point started to become transparent, gradually making the whole colony transparent (2). Twort concluded that an infectious, filterable agent that could kill bacteria and multiply itself caused the transparent transformation (2, 5). Unfortunately, Twort could not proceed with his work because of financial difficulty (1). In 1917, d’Herelle independently rediscovered the phenomenon while working with patients with haemorrhagic dysentery. d’Herelle isolated ‘anti-Shiga microbes’ from the stools of patients recovering from shigellosis by filtering the stools after incubation for 18 h. This active filtrate caused the stasis, death and lysis of *Shigella* species when added to cell suspension cultures. d’Herelle viewed this to be due to a living non-visible virus existing as a parasite of bacteria (6, 7). He proposed the name ‘bacteriophage’, which means ‘bacteria to eat’ in the Greek language (1). Immediately thereafter, bacteriophages have been tested in animals and humans for the treatment of bacterial infections (1, 8, 9). d’Herelle (10) found that bacteriophages were active against not only dysentery but also *Shigella* sp., *Salmonella* sp., *Escherichia coli*, *Proteus vulgaris*, *Vibrio cholerae*, *Yersinia* species, *Corynebacterium diphtheriae* and *Bacillus subtilis*.

d’Herelle initiated the use of bacteriophages in clinical practice and have published many trial experiences (6). In a few earlier animal trials, Davison (8) injected large amounts of bacteriophage in a rabbit and was convinced of its safety. Using the d’Herelle method to isolate bacteriophages, Davison then tested it in 12 paediatric patients with dysentery, of whom five survived from the deadly disease. Shortly thereafter, McKinley (11) injected bacteriophage directly in infected tissues and found that the patients improved with the treatment. In fact, d’Herelle (10) reported that bacteriophage can be delivered as an intravenous or intramuscular injection in staphylococcal septicaemia, streptococcal septicaemia, typhoid fever and cholera. The research interest in and clinical use of bacteriophages waned after the discovery of antibiotics. However, with the increasing trend in the incidence of infection due to multidrug-resistant (MDR) organisms, interest in bacteriophage therapy has been revived.

### The Use of Bacteriophages in the Treatment of Multidrug-Resistant Acinetobacter Infection

MDR *Acinetobacter baumannii* represents a great challenge in clinical practice worldwide. The non-fermenting Gram-negative bacteria of the genus *Acinetobacter* has emerged as one of the most problematic causative agents of healthcare-associated infections worldwide (12). Organisms naturally occurring in the environment, such as *Acinetobacter*s, can be part of the human skin flora. Among *Acinetobacter* sp., *A. baumannii* is the most important pathogen and the most resistant to antimicrobial agents (12, 13). Wide-spectrum clinical infections exist, ranging from less severe urinary tract and wound infections to severe pneumonia, bloodstream infection and meningitis (12–15).

The primary difficulty in treating *Acinetobacter* infections is due to its acquired antimicrobial resistance. The rapid global emergence of *A. baumannii* strains that are resistant to virtually all available antimicrobial agents is quite alarming (13, 15). *Acinetobacter* expresses almost all resistance mechanisms, including reduced number and size of porins, the presence of active efflux systems, penicillin-binding protein mutation and the production of all classes of β-lactamase. Its genomic resistance island is comprised of 45 resistance genes (13). As *Acinetobacter* has become more resistant to antimicrobial agents, with some strains resistant to all available agents, the treatment options for *Acinetobacter* infection have become limited. One remaining potential agent is bacteriophage.

### Acinetobacter Bacteriophage Characterisation and Genomic Analysis

The major limitation of the findings of earlier publications on *Acinetobacter* bacteriophages is that the genus *Acinetobacter* has undergone a long history of taxonomic change (14). Moreover, most publications on bacteriophages were not in the English language. Although Ackermann et al. (16) reported that 29 *Acinetobacter* bacteriophages have been described in the literature since 1966, we found that the earliest report in English on specific bacteriophages against *Acinetobacter* was that by Twarog and Blouse (17), which was published in 1968. Bacteriophages against *Acinetobacter* infection have been classified into eight phage species belonging to the *Myoviridae*, *Siphoviridae* and *Podoviridae* families (16). Most earlier publications have indicated that *Acinetobacter* bacteriophages were mainly used for genetic studies and strain typing of the *Acinetobacter* sp. An earlier work on the therapeutic use of *Acinetobacter* bacteriophages was conducted by Soothill (18). Soothill reported the use of the phage BS46 to treat intra-peritoneal *Acinetobacter* infection in a mouse model. However, a safety study and complete genomic analysis of this bacteriophage were published in 2016 (19) and 2020 (20), respectively. Besides Soothill’s work, only two other studies on the behaviour of bacteriophages against *Acinetobacter* infection, which were by Herman and Juni (21) and Klovins et al. (22), were found. Herman and Juni identified bacteriophage P78 as lysogenised to *A. calcoaceticus*, whereas Klovins et al. sequenced the nucleic acid of the phage AP205, which inconsistently caused lysis in *Acinetobacter* sp.

In 2010, the search for candidate therapeutic/disinfectant agents to control or treat nosocomial infections caused by MDR *Acinetobacter* was commenced. The aims of the in vitro works were mainly to screen, characterise and sequence the genomes of lytic bacteriophages. The bacteriophage screening and characterisation are presented in Table 1. So far, more than 30 genomic analyses of bacteriophages that infect *Acinetobacter* species have been performed. These bacteriophages are members of the order Caudovirales and include *Myoviridae*, the φKMV-like group (of the *Podoviridae* family), other *Podoviridae* and *Siphoviridae* families. The only bacteriophage outside the order *Caudovirales* is ϕkm18p, which belongs to the *Corticoviridae* family (23). The genome sizes of the *Acinetobacter* bacteriophages identified were 31 kb–234 kb, with 14–402 open reading frames (ORF) and 33.5%–45.5% G+C contents (Table 2).

### Cell Culture Studies

Experiments in cell culture play two major roles: i) to determine the cytotoxic effect of the bacteriophages and ii) to preliminarily evaluate the bacteriophage activity in *Acinetobacter*-infected cells. For example, the potential cytotoxicity of the phage PD6A3 was evaluated using human embryonic kidney cell 293 (HEK293T) and a human monocytic cell (THP-1). The viable cells were similar to the control cells after 24 h of exposure to bacteriophage, indicating that no significant cytotoxicity (42). Yin et al. (68) used HeLa cells (from cervical cancer cells) and THP-1 cells to investigate the toxicity of the phage Abp1. They found that the bacteriophage had no toxic effect of the cells and suggested that it is safe for use in vivo.

On the other hand, the preliminary activity of bacteriophage was investigated in a living cell infection model by Yin et al. (68) and in a HeLa cell infection model by Wang et al. (69). Yin et al. (68) found that the phage Abp1 rescued the *Acinetobacter* HeLa-infected cells when administered 2 h after the onset of infection. The survival of the cells was almost identical to that of the uninfected negative controls. On the other hand, Wang et al. (69) found that φm18p phage therapy did not significantly affect the survival of macrophage cells infected with *Acinetobacter*. However, increased concentration of the phage was associated with increased bacterial clearance.

### Animal Study

Only a few studies have been published on bacteriophage testing on invertebrate *Acinetobacter* infections. Jeon et al. (45) conducted a study using a *Galleria mellonella* larvae infection model before conducting animal studies. They found that 90% and 100% of the untreated larvae were dead at 16 h and 24 h, respectively. At a multiplicity of infection of 100, the phage-treated larval group showed a significantly higher survival rate of 50% at 48 h. In another study, Grygorcewicz et al. (54) observed similar results when using the phage AGC01 to treat the same *G. mellonella* larva infection model.

So far, we found 14 murine animal trials on the use of bacteriophage in an *Acinetobacter* sepsis model. A summary of the studies is shown in Table 3.

### Clinical Study

Two published clinical case reports pertained to the use of bacteriophages in the treatment of *Acinetobacter* infections (75, 76). Schooley et al. (75) reported on the use of bacteriophage cocktails to treat necrotising pancreatitis complicated by an MDR *A. baumannii*-infected pancreatic pseudocyst in a 68-year-old diabetic man. A cocktail containing four of the anti-*A. baumannii* bacteriophages (AB-Navy1, AB-Navy4, AB-Navy71 and AB-Navy97) was administered percutaneously to the pseudocyst on day 109 of infection after the patient developed septic shock due to failure of the previous treatment and the unavailability of any active antimicrobial agents. The intravenous therapy was continued, as the patient could tolerate the topical infusion. However, the phage therapy was discontinued for a while because of other nosocomial infections and resumed after the other infections subsided. The selection of an appropriate therapeutic cocktail was based on the bacteriophage activity in *A. baumannii* isolated from pseudocysts. The bactericidal activity was more pronounced when a cocktail of active bacteriophages was used compared with when individual bacteriophages were used separately. After completing 8 weeks of bacteriophage therapy, the patient was discharged home on day 245 and subsequently returned to work (75).

The second case of bacteriophage therapy was reported by LaVergne et al. (76) in a 77-year-old who developed post-operative cerebritis due to MDR *A. baumannii*. The bacteria were resistant to virtually all antibiotics; however, the antibiotic combination test indicated that colistin with rifampicin and colistin with azithromycin approached synergy. After failure of the combination therapies, the patient was treated with a five-bacteriophage cocktail therapy. The selection of bacteriophages was based on the activity against the *A. baumannii* strain found in the patient. Although the patient’s general condition was not remarkably improved, the infections in the craniotomy site and skin flap healed well. However, the patient’s family decided to withdraw the medical care and the patient died on hospital day 20 while on day 8 of phage therapy (76).

### Environment Study

The potential usefulness of bacteriophages as environmental biocontrol agents for MDR *A. baumannii* has attracted great interest. Table 4 shows two important studies published specifically for this purpose.

### Limitations of the Use of Bacteriophages in Clinical Practice

As mentioned earlier, bacteriophages infect specific hosts. These viruses do not commonly cross the host species. In a particular species, their activities are limited to specific strains (Table 1), making it difficult to identify a generic bacteriophage for the treatment of *Acinetobacter* infections. Schooley et al. (75) and LaVergne et al. (76) performed susceptibility tests prior to the selection of the bacteriophage for treatment. However, this method is not practical for all centres, especially in developing countries without virology laboratory support.

Temperate bacteriophages might also enter lysogeny to maintain a long-term relationship with their host bacteria (53). For the own benefit, bacteriophages might not lyse the bacteria, thus producing no significant bactericidal effect for clinical application. These bacteriophages that follow a lysogenic cycle can cause harm instead of clinical improvement to the patient.

To overcome the limited host-range and unpredicted bactericidal activities of bacteriophages, many recent studies have focused on bacteriophage components such as endolysin and depolymerase. More than 10 recent publications have reported on the activity of endolysin from *Acinetobacter* bacteriophage. The host spectrum of endolysin is mostly wider than that of the parent bacteriophage, with some crossing bacterial species (50, 79, 80). On the other hand, depolymerase has been used as an anti-capsular polysaccharide (49, 66). Besides its antibacterial application, this agent can be used as an anti-virulent. Some previously published animal trials using phage components have indicated the potential role of this compound as an antimicrobial agent (42, 49, 79).

## Conclusion

Although bacteriophages have been discovered more than a century ago, research on their potential clinical usefulness against MDR *Acinetobacter* is still in its infancy. Besides the limited number of lytic bacteriophages discovered, only less than 20 reported murine animal trials and two clinical case reports exist in the literature. Further work is needed to develop a bacteriophage cocktail that can infect most MDR *Acinetobacter* strains while maintaining their stability and performance. At the same time, bacteriophage components such as endolysin and depolymerase can be exploited in new antibiotic development research.

## Figures and Tables

**Table 1:** Screening and characterisation of bacteriophage that infect *Acinetobacter* sp.

No.	Year (Ref.)	Phages name	Viral family	Adsorption rate	Latent period* (min)	Burst size@ (PFU/cell)	Host range# (infection/tested strains)
1	2010 (24)	φAB2	*Podoviridae*	> 99% within 8 min	< 10	200	25/127
2	2010 (25)	AB1	Siphoviridae	~90% within 10 min	18	409	1/5
3	2011 (26)	Abp53	*Myoviridae*	99% within 6 min	10	150	7/26
4	2012 (27)	ZZ1	*Myoviridae*	rapidly adsorbed	9	200	3/22
5	2012 (28)	AP22	*Myoviridae*	> 99% within 5 min	40	240	89/130
6	2012 (29)	AB7-IBB1	*Siphoviridae*	> 99 % within 5 min	30	125	23/39
7	2012 (23)	ϕkm18p	Corticoviridae	> 99% within 30 min			15/34
8	2012 (30)	AB7-IBB2	*Podoviridae*	> 99 % within 4 min	25	22	19/39
9	2013 (31)	Abp1	*Podoviridae*		10	350	
10	2014 (32)	AbaM-IME-AB2	*Myoviridae*	> 99% within 9 min	20	62	
11	2014 (33)	ØABP-01	*Podoviridae*		15	110	12/12
12	2014 (33)	ØABP-02	*Myoviridae*		20	120	6/12
13	2014 (33)	ØABP-04	*Myoviridae*		20	150	6/12
14	2018 (39)	AbaM_Acibel004	*Myoviridae*	85% within 15 min	27	125	21/28
15	2014 (34)	AbaP_Acibel007	*Podoviridae*	95% within 10 min	21	145	17/28
16	2016 (35)	GEC_Ab-M-G7	*Myoviridae*	91.1% within 7 min	20	120	136/200
17	2016 (36)	Bφ-C62	*Myoviridae*	80% within 1 min	20	76	16/45
18	2017 (37)	AbaS_Loki	*Siphovirus*	~80% within 5 min	40	43	36/38
19	2017 (38)	AJO1@	*Podoviridae*		30	51.2	
20	2018 (39)	PBAB08	*Myoviridae*	Complete lysis in 25 min	~10	215	5/14
21	2018 (39)	PBAB25	*Myoviridae*	Complete lysis in 25 min	~10	630	1/14
22	2018 (40)	KARL-1	*Myoviridae*	> 99% in 12 min	30	39	21/27
23	2018 (41)	SH-Ab15519	*Podoviridae*	90% within 10 min	10	60	8/48
24	2019 (42)	PD-6A3	*Podoviridae*	> 90% within 5 min	20	129	179/552
25	2019 (43)	Ab105-2ϕΔCI	*Siphoviridae*		30	32	5/20
26	2019 (44)	AB1801	*Siphoviridae*	> 80 % within 10 min	20	212	7/10
27	2019 (45)	Bφ-R2096	*Myoviridae*	95% within 5 min	50	142	17/40
28	2019 (46)	ϕAbp2	*Myoviridae*		15	222	16/60
29	2019 (47)	AJO2	*Podoviridae*		30	78.1	
30	2019 (48)	AbaS_D0	*Siphoviridae*		40	39	18/18
31	2020 (49)	IME285	*Myoviridae*		10	450	25/49
32	2021 (50)	AbaP_D2	*Podoviridae*		20	80	6/48
33	2020 (51)	AbTJ	*Podoviridae*		90	70	5/5
34	2020 (52)	TAC1	*Myoviridae*		15	454	66% of the tested strains
35	2020 (53)	Aba01, Aba02 and Aba03 (similar strain)	*Siphoviridae*	> 98% within 1 min			2/21
36	2020 (54)	AGC01	*Podoviridae*	99% within 5 min	20	317	93/185
37	2021 (55)	øFG02	*Myoviridae*		40	15	1/9
38	2021 (55)	øCO01	*Myoviridae*		~41	30	3/9

Notes:

* Latent period defined as the time interval between adsorption and the beginning of the first burst;

@ Burst size is the number of bacteriophages produced per infected bacterium;

# The tested strain among the host specific species (mostly of *A. baumannii*); PFU = plague-forming unit

**Table 2:** Genetic studies on bacteriophages that infect *Acinetobacter* spp

No.	Year (Ref.)	Phages name	Viral family	Nucleic acid	Genome size (kb)	ORF/CDS#	G+C contents (%)
1	2011 (56)	φAB1	φKMV-like virus	Linear dsDNA	41	46	39.08
2	2011 (26)	Abp53	Myoviridae	dsDNA	95		
3	2012 (57)	YMC/09/02/B1251 ABA BP	Podoviridae	Circular dsDNA	45	62	39.05
4	2012 (29)	AB7-IBB1	*Siphoviridae*		45		
5	2012 (23)	ϕkm18p	*Corticoviridae*	dsDNA	45		
6	2012 (58)	phiAC-1$	*Myoviridae*	Circular dsDNA	43	82	38.5
7	2012 (27), 2014 (59)	ZZ1	*Myoviridae*	dsDNA	166	402	34.3
8	2012 (28)	AP22	*Myoviridae*	dsDNA	46		probably has low G+C content
9	2013 (31)	Abp1	φKMV-like virus *	Linear dsDNA	42	54	39.15
10	2014 (32)	AbaM-IME-AB2	*Myoviridae*	dsDNA	44	82	37.5
11	2015 (60)	Bφ-R3177	*Siphoviridae*	Linear dsDNA	47	14	39.83
12	2015 (61)	AB3	φKMV-like virus *	dsDNA	31	97	39.18
13	2016 (62)	LZ35	*Myoviridae*	dsDNA	44	83	37.95
14	2016 (63)	AbaM_ME3	*Myoviridae*	DNA	234	326	40
15	2016 (36)	Bφ-C62	*Myoviridae*	Linear dsDNA	44	84	37.6
16	2016 (64)	φAB6	*Podoviridae*	dsDNA	41	46	39
17	2017 (37)	AbaS_Loki	*Siphovirus*	dsDNA	41	51	44.35
18	2017 (38)	AJO1@	*Podoviridae*	Linear dsDNA	41	54	
19	2018 (40)	KARL-1	*Myoviridae*		166	115	36.79
20	2018 (41)	SH-Ab15519	*Podoviridae*	Linear dsDNA	40	50	39.5
21	2018 (39)	PBAB08	*Myoviridae*	Linear dsDNA	42	76	
22	2018 (39)	PBAB25	*Myoviridae*	Linear dsDNA	40	70	
23	2019 (48)	AbaS_D0	*Siphoviridae*		43	55	45.48
24	2019 (44)	AB1801	*Siphoviridae*	dsDNA	32		
25	2019 (65)	fHyAci03	*Myoviridae*		165	255	36.8
26	2019 (45)	Bφ-R2096	*Myoviridae*	Linear dsDNA	59	162	
27	2019 (46)	ϕAbp2	*Myoviridae*	Circular dsDNA	45	88	37.84
28	2019 (47)	AJO2@	*Podoviridae*	Linear dsDNA	38	58	41
29	2019 (66)	IME200		DNA	41	54	39.3
29	2019 (42)	PD-6A3	*Podoviridae*		42		38.48
30	2019 (48)	AbaS_D0	*Siphoviridae*	Circular dsDNA	43	55	45.48
31	2020 (49)	IME285	*Myoviridae*	dsDNA	45	83	37.9
32	2020 (52)	TAC1	*Myoviridae*	Linear dsDNA	102	161	37.5
33	2020 (50)	AbaP_D2	*Podoviridae*		40	47	39.23
34	2020 (20)	BS46	*Myoviridae*	Linear dsDNA	94	176	33.5
35	2020 (67)	ApiP_XC38&	*Podoviridae*	dsDNA	79	97	39.58
36	2020 (51)	AbTJ	*Podoviridae*	Circular dsDNA	43	62	
37	2020 (54)	AGC01	*Podoviridae*	DNA	41	55	39.50
38	2021 (55)	øFG02	*Myoviridae*		75	255	44.4
39	2021 (55)	øCO01	*Myoviridae*		87		37.5

Notes:

* *Podoviridae* family;

# open reading frames/coding sequence;

$ infect *A. soli*;

@ infect *A. johnsonii*;

& infect *A. pittii*

**Table 3:** Summary of animal trials utilising phage as one of the treatment regimens of Acinetobacter murine infection models

No.	Year (Ref.)	Phage name	Model (*n* each group)	Intervention	Main outcome
1	1992 (18)	BS46	Mouse bacteraemia by intra-peritoneal inoculation (*n* = 5)	Mice divided into five groups: one control and other four of the groups received 0.25 mL of phage in three-fold dilution steps (4 PFU, 12 PFU, 36 PFU and 108 PFU). Simultaneous intraperitoneal injection of bacteria and treatment was done.	As few as 10^2^ particles of a phage protected mice against 5 LD50 of *A. baumanii* and BS46 phage was demonstrated to have multiplied in the mice.
2	2015 (70)		Rats wound infection	Five groups of uncontrolled diabetic rats were used: (i) non-infected (control), (ii) MDR *A. baumannii* and treated with phage, (iii) MDR *A. baumannii*, (iv) MDR *A. baumannii* and treated with colistin and (v) non-infected rats and sprayed with phage (phage control).	A significant decrease in infection, period of epithelisation and wound contraction was observed in the phage-challenged group when compared with antibiotic-treated uncontrolled diabetic rats and the control groups.
3	2016 (36)	Bφ-C62	Mouse pneumonia model by intra-nasal inoculation (*n* = 6)	Mice were divided into six groups: (i) PBS and SM buffer treated, (ii) *A. baumannii* and SM buffer treated, (iii) PBS and phage treated, (iv) *A. baumannii* and phage treated (MOI* = 10), (v) *A. baumannii* and phage treated (MOI = 1), and (vi) *A. baumannii* and phage treated (MOI = 0.1).	Group (iv) exhibited a 100% survival rate. Group (v) and (vi) showed survival rates of 50% and 16%, respectively.
4	2016 (71)	IME-AB2	Mouse pneumonia model by intra-nasal inoculation (*n* = 72)	Study 1. Mice were divided into four groups: (i) *A. baumannii* pneumonia, (ii) *A. baumannii* and phage treated MOI = 10, treatment at 1 h post-infection, (iii) *A. baumannii* and phage treated MOI = 1, treatment at 1 h, (iv) *A. baumannii* and phage treated MOI = 0.1, treatment at 1 h. Study 2. Mice were divided into four groups (i) *A. baumannii* pneumonia, (ii) *A. baumannii* and phage treated MOI = 10, treatment at 1 h post infection, (iii) *A. baumannii* and phage treated MOI = 10, treatment at 4 h and (iv) *A. baumannii* and phage treated MOI = 10, treatment at 24 h.	Intranasal instillation with bacteriophage at MOI = 10, 1 and 0.1 rescued 100%, 70% and 20% of mice, respectively. Administration of MOI = 10 at 1 h, 4 h and 24 h associated with 100%, 60% and 20% survival, respectively. Phage treatments were also reduced bacterial load in the lung.
5	2016 (72)	AB-Navy1 AB-Navy2 AB-Navy3 AB-Navy4	Mouse wound infection (*n* = 20)	Mice were divided into three groups: (i) no treatment, (ii) treated with AB-Navy1 and (iii) cocktail of four phage. Each wound was inoculated with bioluminescent strains *of A. baumannii* (AB5075:lux*)* and a Tegaderm bandage was placed over the wound. A full-thickness wound was created on the dorsal side of each mouse. For phage treatments, mice received an intraperitoneal injection and topically. Treatments were given simultaneously at 4 h, 24 h and 48 h post-infection.	The measured outcomes which indicate phage cocktail treated significantly better than AB-Navy1, both of them better than untreated (i) surgery/infection-associated weight loss, (ii) average radiance of bioluminescent bacteria in the wound bed, (iii) area of the infection as monitored by the area of bioluminescence, (iv) physical wound size and (v) biofilm formation on wound dressings.
6	2018 (68)	Abp1	Mouse wound infection model (*n* = 12)	Three groups: (i) control, (ii) local phage treatment locally and (iii) systemic phage treatment. Two 5.0 mm × 5.0 mm full-thickness wounds were created on the dorsal side of each mouse. The phage treatments were repeated once a day for 7 days after infection. On day-1, -3 and -7, wound sizes were measured and recorded.	At day-3 and 7 post-infection, wound sizes in animals receiving locally applied phage were significantly smaller than in mice receiving either systemically administered phage or no treatment.
7	2018 (68)	Abp1	Mouse bacteraemia by intra-peritoneal inoculation (*n* = 14)	Three groups: (i) control, (ii) phage treated and (iii) polymyxin B treated (*A. baumannii* strain is sensitive to polymyxin B). Phage in PBS or polymyxin B were injected immediately and everyday intraperitoneally. During the observation period, dead mice were dissected immediately to obtain the livers and kidneys. Seven days post-infection, survivors were also sacrificed and dissected.	Group (i) succumbed rapidly. Only one mouse in this group survived the entire 7-day observation period. In contrast, all mice in groups (ii) and (iii) survived the entire 7 days of the observation period. In group (i), livers and kidneys contained high bacterial loads, while groups (ii) and (iii) contained almost no bacteria in these organs.
8	2018 (69)	ϕkm18p	Mouse bacteraemia by intra-peritoneal inoculation (*n* = 10–15)	Two studies to evaluate the survival of mice and 3 h post-infection bacterial burden. Two different strain of mice were infected by MDR *A. baumannii*. The phage was administered intraperitoneally with different MOIs 10, 1, and 0.1 at 10 min (immediate phage therapy) and 1 h (delayed phage therapy) at different inoculation sites. For survival analysis, mice were kept for 2 weeks whereas for bacterial burden study, blood were collected from the orbital areas after 3 h of treatment.	In BALB/c mice, the survival of all three MOIs phage treated were 100%, in contrast to no survival after day 1 in the control group. In C57BL/6 mice, the survival in treated groups were 88%–100% compared to 46% in control group. The bacterial load in control group is significantly higher than treated groups. Interestingly, phage-resistant mutants were isolated from mouse sera after phage therapy.
9	2018 (73)	Cocktail of 64 phages	Mouse bacteraemia by intra-peritoneal inoculation (*n* = 5)	Mice were divided into four groups: (i) therapeutic phage alone, (ii) bacteria alone, (iii) bacteria inoculation with phage treatment after 2 h and (iv) did not receive any kind of injections.	All mice in bacteremic group without treatment died within 4 h of inoculation. All other group survive until 6 weeks.
10	2018 (41)	SH-Ab 15519	Mouse pneumonia model by intratracheal inoculation (*n* = 10)	Study 1: Mice were divided into five groups: (i) control uninfected, (ii) control bacterial infection, (iii) phage rescues MOI = 10, (iv) phage rescue MOI = 1 and (v) phage rescue MOI = 0.1. Study 2: Mice divided into four groups: (i) control uninfected, (ii) control bacterial infection, (iii) phage rescues 1 h and (iv) phage rescue 2 h.	Results showed that treatment with phage at an MOI = 0.1, 1 or 10 significantly increased the survival rate (90%) compared with the non-phage-treated control group (10%). Administering phage in MOI = 10 at 1 h and 2 h resulted in survival rates of 83.33 and 66.67%, respectively.
11	2018 (39)	PBAB08, PBAB25, PBAB68, PBAB80, PBAB93	Mouse pneumonia model by intra-nasal inoculation (*n* = 20)	Mice were divided into four groups; (i) treated with SM buffer only, (ii) infected with MDR *A. baumannii* only, (iii) treated with the phage cocktail only and (iv) infected with *A. baumannii* and treated with the phage cocktail.	Only 15% of group (ii) survived at 7 days post infection compared to 35% of Group (iv). Groups (i) and (iii) remained healthy for the entire experimental period.
12	2019 (48)	vB_AbaS_D0, vB_AbaP_D2	Mouse bacteraemia by intra-peritoneal inoculation (*n* = 10)	Mice were divided into four groups: (i) sterile saline; (ii) phage D2; (iii) phage D0; (iv) the phage cocktail. Two hours post-infection, mice of each group were treated with phage or sterile saline. Forty-eight hours after treatment, blood samples were collected from the caudal veins of all surviving mice. All *A. baumannii* colonies of each mouse were tested for phage resistance. Survival was tracked for 7 days.	Mortality was 100% after 24 h in group (i). Groups (ii), (iii) and (iv) yielded 90%, 50% and 100% survival, respectively, after 36 h. The percentage of phage resistant mutants occurring in the group (ii) was significantly higher than in groups (iii) and (iv).
13	2019 (45)	Bφ-R2096	Mouse pneumonia model (*n* = 6)	Mice were divided into six groups: (i) buffer-only-treatment, (ii) bacteria-only-treatment, (iii) phage-only-treatment, (iv, v and vi) post-infection phage-treatments (MOI = 10, 1 and 0.1, respectively).	Group (ii) all died by day 5 post-infection; High survival rates on day 12 at MOI = 10 (100%), MOI = 1 (60%) and MOI = 0.1 (30%). Moreover, no mice in group (i) or (iii) died and lost weight
14	2019 (42)	PD-6A3	Mouse bacteraemia by intra-peritoneal inoculation (*n* = 10)	Mice were divided into eight group: (i) control bacteraemia, (ii) control endolysisn, (iii) control phage, (iv) endolysin therapy group, (v) endolysin + PD-6A3 phage therapy group, (vi) PD-6A3 phage therapy group, (vii) phage cocktail and (viii) PBS control group.	The survival rate of groups (iv), (v), (vi) and (vii) were 70, 70, 60 and 50%, respectively, higher than the bacterial group (0% survival). White-blood-count counts in all four therapy groups were significantly lower than those in group (i) (*P* < 0.05). The levels of IL-10 and PCT were significantly elevated in sepsis group compared to four therapy groups.
15	2020 (74)	AbArmyϕ1, AbNavyϕ1, AbNavyϕ2, AbNavyϕ3, and AbNavyϕ4	Mouse wound infection	Mice were divided into four group based on their pre-treatment/post-treatment regimen: (i) PBS-PBS (control), (ii) PBS-phage (phage treatment), (iii) phage-PBS (phage prophylaxis) and (iv) phage-phage (phage treatment and prophylaxis). A full-thickness wound was created on the dorsal side and inoculated with bioluminescent strains AB5075:lux. Phages cocktail treatments were administered at 4 h, 24 h and 48 h post-infection.	Group (i) had a high bacterial bioburden 24 h after infection. Bacterial burden did not appear to be altered by prophylactic administration (group (iii)). The treatment regimen of phage mixture (group (ii) was able to promptly reduce bacterial bioburden. Bacterial bioburden of group (iv) intermediary effect between groups (i) and (ii). Similar result with the would size between groups.

Note:

* multiplicity of infection (MOI) is a frequently used term in virology which refers to the number of virions that are added per cell during infection

**Table 4:** The trial using bacteriophages to reduce *Acinetobacter* burden for external use and in environment

Study	Year (Ref.)	Phages name	Interventions	Main outcome
1	2013 (77)	φAB2	The bactericidal effect of three φAB2 preparations: in a liquid suspension, in a paraffin oil-based lotion and in glycerol were tested on *A. baumannii* M3237 suspension and on glass surface.	The addition of φAB2 at a concentration of at least 10^5^ PFU/mL to suspension and 10^8^ PFU/slide to glass slides are associated with > 90% killing of *A. baumannii*. The φAB2 preparation in glycerol is still active after 90 days storage. The φAB2 in 10% paraffin had no activity in the lotion after 1 month of storage.
2	2016 (78)	φAB1 φAB2 φAB6 φAB7 φ4C08 φ8C07 φAB11 φ5C05	Terminal cleaning using standard procedures plus an aerosol with active bacteriophage in the ICU. Eight different single phages were sequentially used according to the phage typing results from each CRAB strain that was clinically isolated from the patient.	The rates of new acquisitions of CRAB in the ICU decreased from 8.57 per 1,000 patient-days in the pre-intervention period to 5.11 per 1,000 patient-days in the intervention period (*P* = 0.0029). The mean percentage of resistant isolates CRAB decreased from 87.76% to 46.07% in the intensive care units (*P* = 0.001). The antimicrobials showed a significant reduction in consumption except imipenem.

Note: CRAB = carbapenem-resistant *Acinetobacter baumannii*
